# Effects of 12-Week Resistance Training on Metabolic and Immune Responses in Normal-Weight and Older Women with Obesity

**DOI:** 10.3390/ijms27177592

**Published:** 2026-08-25

**Authors:** Nicolás Vidal-Seguel, Alexis Sepúlveda-Lara, Juan Carranza-Leiva, Carlos Márquez, Nolberto Huard, Jorge Sapunar, Luis A. Salazar, Sergio Sanhueza, Solange Torres, Estefanía Nova-Lamperti, Gabriel Nasri Marzuca-Nassr

**Affiliations:** 1 Departamento de Ciencias Básicas, Facultad de Medicina, Universidad de La Frontera, Avenida Francisco Salazar 01145, Temuco 4811230, Chile; nicolasenrique.vidal@ufrontera.cl; 2 Programa de Doctorado en Ciencias Morfológicas, Facultad de Medicina, Universidad de La Frontera, Avenida Francisco Salazar 01145, Temuco 4811230, Chile; 3 Departamento de Procesos Terapéuticos, Facultad de Ciencias de la Salud, Universidad Católica de Temuco, Manuel Montt 056, Temuco 4780000, Chile; alexis.sepulveda@uct.cl; 4 Doctorado en Ciencias Mención Biología Celular y Molecular Aplicada, Facultad de Ciencias Agropecuarias, Universidad de La Frontera, Avenida Francisco Salazar 01145, Temuco 4811230, Chile; 5 Departamento de Ciencias de la Rehabilitación, Facultad de Medicina, Universidad de La Frontera, Claro Solar 115, Temuco 4811230, Chile; juan.carranza@ufrontera.cl; 6 Unidad de Kinesiología, Clínica de Medicina Física y Rehabilitación MEDIFIS, Dieciocho de Septiembre 611, Claro Solar 115, Temuco 4780000, Chile; 7 Departamento de Medicina Interna, Facultad de Medicina, Universidad de La Frontera, Claro Solar 115, Temuco 4811230, Chile; carlos.marquez@ufrontera.cl (C.M.); jorge.sapunar@ufrontera.cl (J.S.); 8 Núcleo de Envejecimiento, Vejez y Calidad de Vida, Universidad de Chile, Avenida Libertador Bernardo O’Higgins 1058, Santiago 8330015, Chile; 9 Centro de Biología Molecular y Farmacogenética, Departamento de Ciencias Básicas, Facultad de Medicina, Universidad de La Frontera, Avenida Francisco Salazar 01145, Temuco 4811230, Chile; nolberto.huard@ufrontera.cl (N.H.); luis.salazar@ufrontera.cl (L.A.S.); 10 Centro de Investigación e Innovación del Cáncer, Fundación Arturo López Pérez OECI Cancer Center, José Manuel Infante 805, Santiago 7500000, Chile; 11 Laboratorio de Inmunología Molecular y Traslacional, Departamento de Bioquímica Clínica e Inmunología, Facultad de Farmacia, Universidad de Concepción, Víctor Lamas 1290, Concepción 4070386, Chile; sergiosanhueza@udec.cl (S.S.); enova@udec.cl (E.N.-L.); 12 Laboratorio de Química de Productos Naturales, Departamento de Botánica, Facultad de Ciencias Naturales y Oceanográficas, Universidad de Concepción, Víctor Lamas 1290, Concepción 4030000, Chile; soltorres@udec.cl; 13 Instituto de Ciencias Naturales, Facultad de Medicina Veterinaria y Agronomía, Universidad de las Américas, Campus El Boldal, Av. Jorge Alessandri 1160, Concepción 4090940, Chile

**Keywords:** ageing, obesity, NETosis, cytokines, muscle quality

## Abstract

Ageing and obesity represent major public health challenges and are associated with metabolic dysfunction, chronic inflammation, and loss of skeletal muscle mass. Resistance training (RT) is an effective strategy to increase muscle mass; however, its effects on metabolic and immune parameters in older women with obesity remain unclear. The study aimed to compare the effects of 12-week progressive overload RT programme on metabolic and immune responses in women aged 60–79 years with obesity and normal-weight women. Thirty older women were allocated into two groups according to body composition: women with obesity (OB: 68 ± 4.21 years; body mass index [BMI] = 33.01 ± 2.62 kg/m^2^; body fat percentage = 42.23% ± 2.98%; *n* = 16) and normal-weight women (NW: 66 ± 4.31 years; BMI = 22.60 ± 1.36 kg/m^2^; body fat percentage = 33.11% ± 3.61%; *n* = 14). At baseline, the OB group showed higher serum concentrations of leptin, insulin, triglycerides, and HOMA-IR and lower HDL cholesterol concentrations compared with the NW group, while no differences were observed in circulating cytokines or NETosis. After 12 weeks of RT, fasting glucose decreased only in the OB group (*p* = 0.030). Both groups exhibited reductions in total cholesterol and adiponectin, along with an increase in NETosis. Leptin, insulin, and pro-inflammatory cytokines were associated with lower muscle quality, whereas higher baseline adiponectin concentrations were associated with greater muscle mass gains in the NW group (*p* < 0.05). In conclusion, baseline metabolic differences were observed between normal-weight older women and those with obesity; however, both groups showed changes after 12 weeks of RT in metabolic and immune parameters. Furthermore, higher baseline adiponectin concentrations were associated with greater muscle mass gains in the NW group.

## 1. Introduction

According to the World Health Organization (WHO), the global proportion of individuals aged 60 years and older is projected to increase from 12% to 22% between 2020 and 2030 [1]. In parallel, obesity, recognised as one of the leading causes of morbidity and mortality [2], has a global prevalence of 25.3% among older adults [3]. In this context, both population ageing and obesity represent two of the most critical public health challenges worldwide [4].

Ageing is characterised by a progressive decline in skeletal muscle mass, accompanied by reductions in muscle strength and functional capacity [5], as well as an increased adipose tissue accumulation and redistribution, which contributes to a higher prevalence of obesity [5]. In women, these changes are further exacerbated after menopause due to the decline in oestrogen levels, which promote the loss of skeletal muscle mass, deterioration of muscle quality, reduced muscle strength, and greater accumulation and redistribution of adipose tissue [6]. Consequently, adipose tissue dysfunction is associated with metabolic alterations, including insulin resistance, dyslipidaemia (altered cholesterol levels and high- and low-density lipoproteins) [7], and a chronic low-grade inflammatory state [8], thereby increasing cardiometabolic risk [8,9]. Likewise, adipose tissue dysfunction negatively affects both skeletal muscle mass and muscle quality in older adults [10,11]. Muscle quality refers to a concept encompassing both the morphological and functional characteristics of skeletal muscle [12].

Obesity is associated with hormonal dysregulation, primarily mediated by adipocyte-derived hormones, which play a central role in the vicious cycle of hyperinsulinaemia and chronic inflammation [13]. On the one hand, leptin, a hormone involved in the appetite regulation, exhibits elevated circulating concentrations in individuals with obesity, leading to the development of hypothalamic leptin resistance. This alteration promotes dysregulated food intake, impairs body weight regulation, and contributes to hyperinsulinaemia [14]. Conversely, adiponectin is associated with increased insulin sensitivity and anti-inflammatory effects; however, its circulating concentrations are reduced in individuals with obesity, thereby promoting a pro-inflammatory milieu and increasing cardiometabolic risk [15]. Collectively, elevated leptin and reduced adiponectin concentrations may contribute to skeletal muscle mass loss in older adults [16].

From an immunological perspective, ageing is associated with functional alterations in multiple immune cell populations, including lymphocytes, macrophages, and neutrophils [17]. These changes impair immune system efficiency and promote excessive production of pro-inflammatory cytokines [18], such as tumour necrosis factor alpha (TNF-α), interleukin-1 beta (IL-1β), and interleukin-6 (IL-6) [19,20]. These cytokines contribute to the development of chronic low-grade inflammation [21], which has been associated with an increased risk of type 2 diabetes mellitus [22], cardiovascular diseases [23], insulin resistance [20] and cancer [24]. Furthermore, this inflammatory environment contributes to impaired muscle quality and a reduced anabolic response in skeletal muscle [21,25].

In the context of ageing and obesity, excessive NETosis has emerged as an immune mechanism of increasing interest [26,27]. NETosis is a form of neutrophil cell death characterised by the release of neutrophil extracellular traps (NETs), whose physiological function is to capture and limit the dissemination of pathogens as part of the innate immune response [28,29]. However, older adults have been shown to exhibit reduced NET efficiency and excessive NETosis production in response to sterile inflammatory stimuli [27]. Similarly, in individuals with obesity, elevated circulating concentrations of TNF-α, IL-6, and interleukin-8 (IL-8) have been associated with increased NETosis [30]. This dysregulated immune response is considered to contribute to the development of obesity and obesity-related conditions, such as type 2 diabetes mellitus, atherosclerosis [26] and other cardiometabolic diseases [31,32]. Moreover, excessive NETosis has recently been linked to skeletal muscle damage and impaired muscle regenerative capacity [33,34,35]. To counteract these alterations, RT, widely regarded as the gold standard intervention for increasing skeletal muscle mass [36], has emerged as a non-invasive therapeutic strategy capable of improving metabolic and immune disturbances in older adults. The mechanical stimulus induced by RT not only promotes muscle hypertrophy but also improves skeletal muscle metabolic function [37], making it a key intervention for reducing age-related chronic inflammation [38,39].

The available evidence on the effects of RT on metabolic and immune parameters in older women with different body compositions remains limited and heterogeneous. Most studies have focused on older women without obesity, reporting that RT programmes effectively reduce markers of metabolic syndrome and inflammation [40]. In older women with overweight or obesity, RT has shown positive effects on fasting glucose, total cholesterol, and LDL cholesterol, although no significant changes have been observed in triglycerides or HDL cholesterol [41]. These findings suggest that obesity may attenuate some of the metabolic benefits of RT [42]. From an immunological perspective, RT plays a relevant role in reducing chronic low-grade inflammation [43]. It has been reported that 12 weeks of resistance training significantly decreases the concentrations of TNF-α and IL-6 in women with normal weight and obesity [43].

Despite these advances, studies examining the effects of RT on metabolic and immune parameters in older adults with different body composition profiles remain limited [44,45]. Furthermore, it remains unclear how these alterations relate to morphological and functional parameters of muscle quality, as well as to RT-induced muscle mass gains in older women with and without obesity. Therefore, the aim of the present study was to compare the effects of 12 weeks of an RT programme on metabolic and immune responses in women aged 60–79 years with obesity and age-matched normal-weight women, as well as to investigate the associations between metabolic and immune biomarkers and measures of body composition and muscle quality. We hypothesised that the metabolic and immune responses to a 12-week resistance training (RT) programme would differ between older women with obesity and age-matched normal-weight women and that metabolic and immune biomarkers would be associated with measures of body composition and muscle quality.

## 2. Results

### 2.1. Baseline Characteristics

Baseline characteristics of the participants are presented in Table 1. Significant differences were observed in BMI, muscle mass, fat mass, visceral fat, and waist circumference (*p* < 0.05), with higher values in the OB group compared with the NW group. The prevalence of underlying chronic diseases was comparable between groups for dyslipidaemia and hypertension; however, the prevalence with type 2 diabetes mellitus was significantly higher in the OB group (*p* = 0.033).

### 2.2. Dietary Intake and Physical Activity Levels

Dietary macronutrient intake remained unchanged throughout the intervention, with no significant differences between baseline and post-intervention assessments. Similarly, moderate-intensity physical activity (*p* = 0.585, η^2^ = 0.010), sedentary behaviour (*p* = 0.320, η^2^ = 0.035), and daily step count (*p* = 0.445, η^2^ = 0.045) did not change over the 12-week period. In contrast, vigorous-intensity physical activity increased significantly following the resistance training intervention (time effect: *p* < 0.001, η^2^ = 0.383), reflecting adherence to the prescribed exercise programme [46].

### 2.3. Metabolic Parameters

At baseline, significant differences were observed between the NW and OB groups in serum insulin, HOMA-IR, leptin, HDL cholesterol, and triglyceride concentrations (independent *t*-test, *p* < 0.004; d > 1.136; Table 2). Compared with the NW group, the OB group exhibited higher baseline concentrations of insulin, HOMA-IR, leptin, and triglycerides, as well as lower HDL cholesterol concentrations. Nevertheless, all measured values remained within the clinically normal ranges.

After 12 weeks of RT, a significant reduction was observed in adiponectin concentrations (time effect, *p* = 0.049; η^2^ = 0.131) and total cholesterol (time effect, *p* = 0.047; η^2^ = 0.134). In addition, a significant time × group interaction was detected for fasting glucose (*p* = 0.021; η^2^ = 0.177), with a significant reduction observed exclusively in the OB group (*p* = 0.030). Moreover, the OB group exhibited a greater change (Δ) in glucose concentration compared with the NW group after 12 weeks of RT (*p* = 0.021; Table 3).

### 2.4. Immune Parameters

At baseline, no significant differences were observed between the NW and OB groups in cytokine concentrations or NETosis induction (independent *t*-test, *p* > 0.104; d < 0.615; Table 2).

Following 12 weeks of RT, no significant changes were detected in cytokine concentrations (time effect, *p* > 0.224; η^2^ < 0.052). However, analysis of the time × group interaction revealed significant effects for IL-12 (*p* = 0.008; η^2^ = 0.227) and TNF-α (*p* = 0.040; η^2^ = 0.104), with differences observed exclusively in the post-intervention assessment (*p* < 0.020). Change (Δ; Post − Pre) analysis demonstrated significant between-group differences for IL 12 (*p* = 0.008), with a decrease in the OB group (−0.12 ± 0.23 pg/mL) and an increase in the NW group (+0.14 ± 0.25 pg/mL). Similarly, a significant difference was observed for TNF-α changes values (*p* = 0.040), with a reduction in the OB group (−0.18 ± 0.44 pg/mL) and an increase in the NW group (+0.22 ± 0.56 pg/mL) (Table 4).

Regarding NETosis induction, 12 weeks of RT resulted in a trend toward increased NET formation, reaching statistical significance at 24 h of incubation (time effect, *p* = 0.044; η^2^ = 0.137; Table 4).

### 2.5. Correlation Between Metabolic and Immune Parameters and Muscle Quality

**Figure 1:**
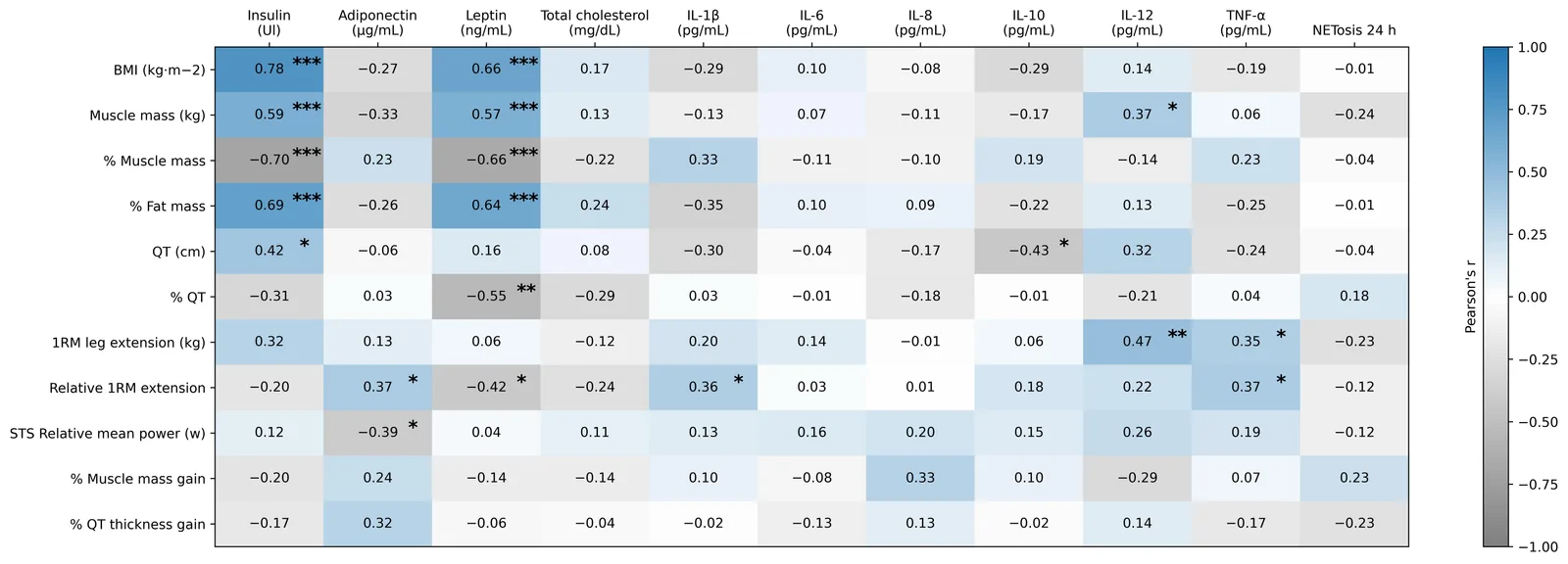
Correlation between metabolic/immune parameters and muscle quality parameters in all participants. IL: interleukin, TNF: tumour necrosis, BMI: body mass index, QT: quadricep thickness; factor; Pearson’s value is presented in each cell; white–blue gradient colour indicates a positive correlation and grey gradient colour indicates a negative correlation. *p* values are reported by * *p* < 0.05, ** *p* < 0.01, *** *p* < 0.001.

**Figure 2:**
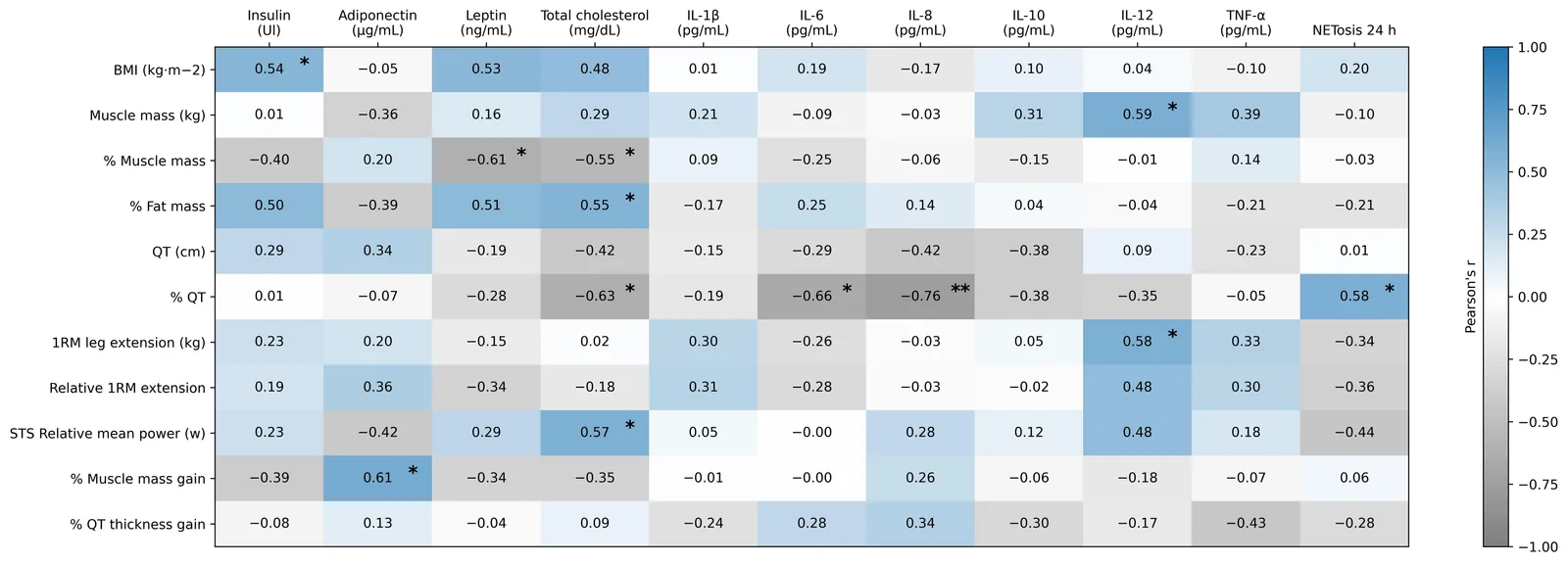
Correlation between biochemical markers and muscle quality parameters in normal-weight women. IL: interleukin, TNF: tumour necrosis, BMI: body mass index, QT: quadricep thickness; factor; Pearson’s value is presented in each cell; white–blue gradient colour indicates a positive correlation and grey gradient colour indicates a negative correlation. *p* values are reported by * *p* < 0.05, ** *p* < 0.01.

**Figure 3:**
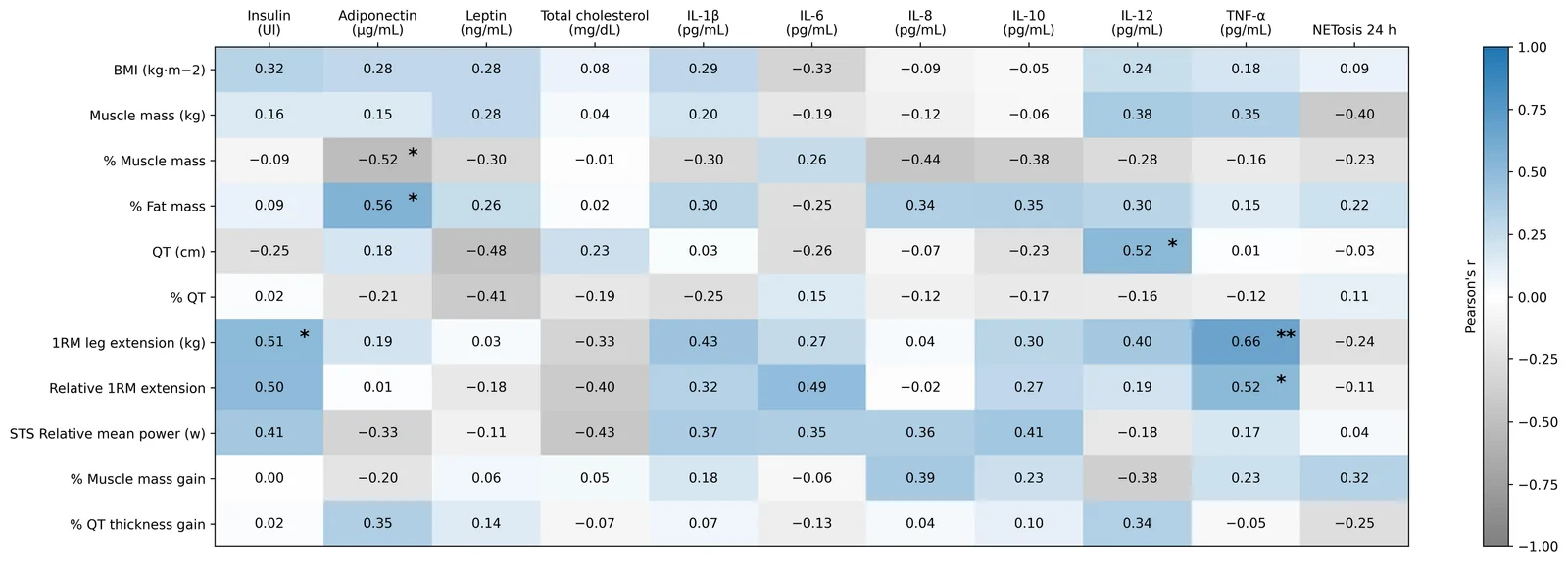
Correlation between biochemical markers and muscle quality parameters in women with obesity. IL: interleukin, TNF: tumour necrosis, BMI: body mass index, QT: quadricep thickness; factor; Pearson’s value is presented in each cell; white–blue gradient colour indicates a positive correlation and grey gradient colour indicates a negative correlation. Values *p* are reported by * *p* < 0.05, ** *p* < 0.01.

Correlations between metabolic and immune parameters and body composition and muscle quality measures are presented for the total sample (Figure 1) and stratified by NW and OB groups (Figure 2 and Figure 3).

From a metabolic perspective, insulin concentration was positively correlated with BMI, muscle mass (kg), percentage fat mass, and quadricep thickness (QT, cm), and negatively correlated with percentage muscle mass (*p* < 0.05; Figure 1). Group-specific analyses revealed a positive correlation between insulin and BMI in the NW group (*p* = 0.049; Figure 2) and between insulin and 1RM leg extension in the OB group (*p* = 0.045; Figure 3).

In the total sample, adiponectin concentration was positively correlated with relative 1RM leg extension (*p* = 0.038) and negatively correlated with STS relative mean power (*p* = 0.026). When analysed by group, participants in the OB group showed a positive correlation between adiponectin and percentage fat mass (*p* = 0.025) and a negative correlation with percentage muscle mass (*p* = 0.038).

Leptin concentration was positively correlated with absolute muscle mass, percentage fat mass, and BMI, and negatively correlated with percentage muscle mass, relative quadricep thickness, and relative 1RM leg extension (*p* < 0.001; Figure 1). In group-specific analyses, participants in the NW group showed a negative correlation between leptin and percentage muscle mass (*p* = 0.019).

Cholesterol concentration exhibited significant correlations exclusively in the NW group, showing positive associations with percentage fat mass (*p* = 0.041) and STS relative mean power (*p* = 0.033) and negative associations with percentage muscle mass (*p* = 0.042) and percentage QT (*p* = 0.015).

From an immune perspective, selective correlations were observed between cytokines (IL-1β, IL-6, IL-8, IL-10, IL-12, and TNF-α) and body composition and muscle quality parameters (Figure 1, Figure 2 and Figure 3). In group-specific analyses, one of the most notable findings was observed in the NW group, where IL-6 and IL-8 concentrations were negatively correlated with percentage QT (*p* < 0.010), indicating that higher pro-inflammatory cytokine concentrations were associated with lower quadricep muscle quality. No significant associations were observed between cytokine concentrations and of NETosis induction (*p* > 0.522).

Finally, in the overall sample, no significant correlations were identified between metabolic or immune parameters and muscle mass gain after 12 weeks of RT (*p* > 0.05; Figure 1). However, when analysed by group, participants in the NW group showed a positive correlation between baseline adiponectin concentration and percentage muscle mass gain, such that higher baseline adiponectin concentrations were associated with greater muscle mass gains after 12 weeks of RT (*p* = 0.020; Figure 2).

## 3. Discussion

The present study aimed to compare the effects of 12 weeks of an RT programme on metabolic and immune responses in women aged 60–79 years with obesity and age-matched normal-weight women. The main findings showed that, at baseline, women with obesity exhibited significant differences in metabolic parameters, but not in immune markers, compared with normal-weight women. Following the intervention, RT induced selective improvements in metabolic parameters, including reductions in total cholesterol and fasting glucose, along with a decrease in adiponectin concentrations and an increase in NETosis induction at 24 h of incubation. Finally, correlation analyses revealed significant associations between immune and metabolic biomarkers and measures of body composition and muscle quality in older women with and without obesity.

The baseline metabolic profile differed between older women with obesity and normal-weight older women and was consistent with the metabolic alterations typically associated with obesity. Adipose tissue dysfunction, characterised by adipocyte hypertrophy, ectopic fat accumulation, and dysregulated adipokine secretion, disrupts metabolic homeostasis by promoting increased circulating free fatty acids, hyperleptinaemia, and insulin resistance [47,48]. Notably, despite these between-group differences, all metabolic parameters remained within clinically normal reference ranges. This metabolic profile suggests that the women with obesity included in this study may be classified as having a metabolically healthy obesity phenotype characterised by excess adiposity in the absence of overt metabolic dysfunction [49]. Nevertheless, as this phenotype is not static and may progress to a higher cardiometabolic risk state with ageing and increasing adiposity, these findings should be interpreted with caution.

In contrast to previous studies, no differences in the baseline inflammatory profile were observed between older women with obesity and normal-weight older women. This finding differs from evidence describing obesity as a pro-inflammatory condition characterised by immune cell infiltration into adipose tissue and increased production of inflammatory mediators [50,51]. However, current evidence recognises obesity as a heterogeneous condition with substantial variability in its metabolic and immunological manifestations, as recently acknowledged by an international expert commission that proposed a redefinition of its diagnosis and classification [52]. In this context, our findings are consistent with those reported by Ashraf et al. (2018), who likewise found no significant differences in circulating IL-6 and TNF-α concentrations between individuals with and without obesity [53]. Furthermore, cytokine concentrations observed in our cohort were within the reference ranges reported for healthy young women assessed by flow cytometry [54]. This suggesting the absence of overt systemic inflammation in the participants included in this study. Regarding NETosis, evidence in older women remains limited. Nevertheless, our research group previously demonstrated greater NETosis induction in older than in younger men [55], suggesting that ageing may play an important role in the regulation of this innate immune mechanism. This observation contrasts with previous evidence linking NETosis to obesity, highlighting the complexity of the factors regulating this process [26]. Therefore, further studies are warranted to determine the relative contributions of ageing and body composition to the regulation of NETosis.

Physical exercise is particularly relevant in the context of ageing and obesity, particularly in older women, as both processes accelerate musculoskeletal deterioration [56,57]. During this stage of life, the decline in oestrogen levels associated with menopause contributes to the progressive loss of muscle mass, strength, and muscle quality, increasing vulnerability to sarcopenia and functional limitations [6]. In this context, RT has been widely recognised as an effective intervention to counteract the detrimental effects of ageing on the musculoskeletal system. In addition to improving muscle mass, strength, and physical performance in older adults [36], RT also induces favourable adaptations in the morphological and functional properties of muscle quality in both normal-weight and women with obesity [46]. These benefits are attributed, at least in part, to the mechanical stimulus provided by RT, which not only promotes muscle hypertrophy but also improves the metabolic function of skeletal muscle [37], establishing RT as a key strategy for promoting healthy ageing [38,39]. However, although its benefits for musculoskeletal function are well established, evidence regarding its effects on metabolic and immune parameters in older women with different body composition profiles, as well as their relationship with muscle quality, remains limited.

The findings of the present study suggest that resistance training (RT) exerts beneficial effects on glycaemic control and lipid profile in older women, particularly those with obesity. These findings differ from those reported by de Seixas et al. (2025), who observed improvements in muscle strength but no changes in glucose or lipid profile after 12 weeks of RT in older men without obesity [58]. In contrast, a recent systematic review and meta-analysis by Liu et al. (2025) concluded that RT significantly reduces fasting glucose, glycated haemoglobin, total cholesterol, and LDL cholesterol in older adults with overweight and obesity [41]. These discrepancies may be explained, at least in part, by differences in training programme characteristics. Whereas de Seixas et al. employed a lower-frequency, lower-intensity protocol (two sessions per week at 50% of one-repetition maximum [1RM]), the present study implemented a higher-volume, higher-intensity programme (three sessions per week at 60–80% of 1RM). Consistent with this interpretation, available evidence suggests that higher-volume and higher-intensity RT protocols elicit more favourable metabolic adaptations, including improvements in glycaemic control, lipid profile, and body composition [59].

At the hormonal level, the absence of significant changes in leptin concentrations following 12 weeks of RT (Table 3) is consistent with the available evidence, which has reported heterogeneous responses of this adipokine to exercise [60]. Conversely, a significant reduction in adiponectin concentrations was observed, consistent with the findings of Memelink et al. (2024), who reported decreased adiponectin levels following 13 weeks of RT (60–80% of one-repetition maximum [1RM]) combined with high-intensity interval training in adults with obesity and type 2 diabetes [61]. Although reduced adiponectin levels have traditionally been associated with increased inflammation, insulin resistance, and cardiometabolic risk, the interpretation of this adipokine in older adults and individuals with obesity is complex [62]. Notably, clinical studies have reported an association between elevated adiponectin concentrations and an increased risk of mortality, cardiovascular disease, end-stage renal disease, type 2 diabetes, and stroke, a phenomenon commonly referred to as the adiponectin paradox [63,64,65]. Preclinical studies have proposed two main mechanisms to explain the paradoxical elevation of adiponectin concentrations. One mechanism attributes this phenomenon to reduced hepatic and renal clearance of adiponectin, whereas the other suggests that adipose tissue inflammation impairs its processing and biological function, promoting the release of less biologically active forms [66,67]. Within this framework, the reduction in adiponectin observed following RT may represent a specific training-induced adaptation, although this interpretation should be approached with caution given the complex regulation of this adipokine. Furthermore, the absence of concomitant changes in the inflammatory markers assessed (IL-1β, IL-6, IL-8, IL-10, IL-12, and TNF-α), consistent with the findings reported by Tan et al. (2025) in postmenopausal women with overweight and obesity undergoing RT [68], suggests that an isolated change in adiponectin was not necessarily accompanied by a detectable alteration in systemic inflammatory status.

A notable finding of the present study was the increase in serum-induced NETosis observed after 24 h of incubation following RT. Because NETosis was assessed using neutrophils isolated from a healthy young donor and incubated with serum obtained from the participants, this finding reflects an enhanced capacity of circulating humoral factors to induce NETosis rather than intrinsic changes in neutrophil function in the participants. NETosis is an essential innate immune defence mechanism; however, excessive or sustained NET formation has been associated with ageing, obesity, metabolic disorders, and chronic inflammatory diseases [27,32]. Therefore, the biological significance of the observed increase remains uncertain.

One possible explanation is that RT altered circulating factors involved in the regulation of NETosis that were not evaluated in the present study, including metabolites, reactive oxygen species (ROS), complement proteins, or other inflammatory mediators beyond the cytokines analysed [69]. NET formation is largely driven by ROS, which can be generated through both NADPH oxidase (NOX)-dependent and mitochondrial pathways, giving rise to NOX-dependent and NOX-independent NETosis, respectively. In the NOX-dependent pathway, ROS promote chromatin decondensation and the subsequent release of NETs [70]. In addition, recent evidence suggests that complement activation, particularly through C3a and C5a signalling, can enhance ROS generation and amplify NET formation, highlighting the close interplay between oxidative stress and humoral immune pathways in the regulation of NETosis [69]. Accordingly, the increase in serum-induced NETosis observed following RT may reflect alterations in systemic redox homeostasis and complement activity, thereby increasing the NETosis-inducing capacity of circulating humoral factors.

This interpretation is supported by the absence of concomitant changes in circulating inflammatory cytokines (IL-1β, IL-6, IL-8, IL-10, IL-12, and TNF-α), suggesting that the enhanced NETosis-inducing capacity of the serum was not accompanied by detectable systemic inflammation. This hypothesis may be particularly relevant in the context of obesity and type 2 diabetes mellitus, both of which are characterised by chronic oxidative stress and increased production of reactive oxygen species (ROS) [71,72]. Given the central role of ROS in NET formation, alterations in systemic redox homeostasis may substantially influence the ability of circulating humoral factors to induce NETosis. Future studies should therefore investigate additional serum components involved in NETosis regulation, including markers of oxidative stress and complement activation, to better understand the mechanisms underlying the increased serum-induced NETosis observed following RT.

Although acute exercise has been shown to transiently induce NET formation as part of the physiological response to exercise-induced stress [73,74], the effects of chronic exercise training on circulating factors that regulate NETosis remain poorly understood. In contrast to our findings, previous studies from our group demonstrated that 12 weeks of high-intensity interval training (HIIT) reduced the serum-induced NETosis response in older men [55], and Ondracek et al. (2022) reported that eight months of RT reduced NETosis-related markers in adults [75]. These discrepancies may be explained by differences in exercise modality, intervention duration, participant characteristics, and the methodologies used to evaluate NETosis. Collectively, these findings suggest that the effects of exercise training on the humoral regulation of NETosis are complex and remain to be fully elucidated.

Finally, although resistance training research has traditionally focused on the quantity of muscle mass, the concept of muscle quality, which integrates both morphological and functional characteristics, has gained increasing relevance in ageing research. In this context, the associations observed between metabolic and immune biomarkers and parameters of body composition and muscle quality suggest that the metabolic and inflammatory milieu may be related to muscle health in older adults. However, given the relatively small sample size, these correlation analyses should be considered exploratory and interpreted as hypothesis-generating rather than evidence of causal biological mechanisms. Consistent with previous studies, hyperinsulinaemia and hyperleptinaemia have been associated with greater adiposity and impaired muscle health during ageing [76,77,78,79,80]. In particular, the inverse association between leptin concentrations and skeletal muscle percentage, quadricep thickness, and relative maximal knee extension strength suggests that elevated leptin concentrations are associated with lower skeletal muscle quantity and quality.

From an immunological perspective, the inverse associations between the pro-inflammatory cytokines IL-6 and IL-8 and quadricep thickness are consistent with previous evidence linking chronic low-grade inflammation to age-related muscle deterioration. These findings are consistent with those reported by Grosicki et al. (2020), who observed an inverse relationship between IL-6 concentrations and muscle quality in older adults [81]. Likewise, the positive association between baseline adiponectin concentrations and gains in quadricep thickness following 12 weeks of RT in the normal-weight group suggests that a more favourable metabolic profile may be associated with a greater adaptive response to exercise. Given the insulin-sensitising and anti-inflammatory properties attributed to adiponectin [15], higher baseline concentrations may reflect a metabolic environment that is more permissive to muscle remodelling. However, the relationship between adiponectin and skeletal muscle remains controversial and requires further investigation.

Interestingly, the association between adiponectin and skeletal muscle mass differed according to obesity status. Whereas higher adiponectin concentrations were associated with a higher percentage of skeletal muscle mass in older women with normal weight, the opposite association was observed in women with obesity. This apparent discrepancy may be related to the so-called adiponectin paradox, whereby elevated adiponectin concentrations do not necessarily indicate a favourable metabolic profile but may instead represent a compensatory response to chronic inflammation, metabolic stress, or impaired tissue function [62,66,67]. Therefore, the observed associations between adiponectin and muscle-related outcomes appear to vary according to metabolic status. Nevertheless, these findings should be interpreted with caution because the correlation analyses were exploratory and do not establish causal biological relationships. These findings should be confirmed in larger prospective studies to determine whether the observed associations reflect underlying biological mechanisms.

## 4. Materials and Methods

### 4.1. Participants and Study Design

**Figure 4:**
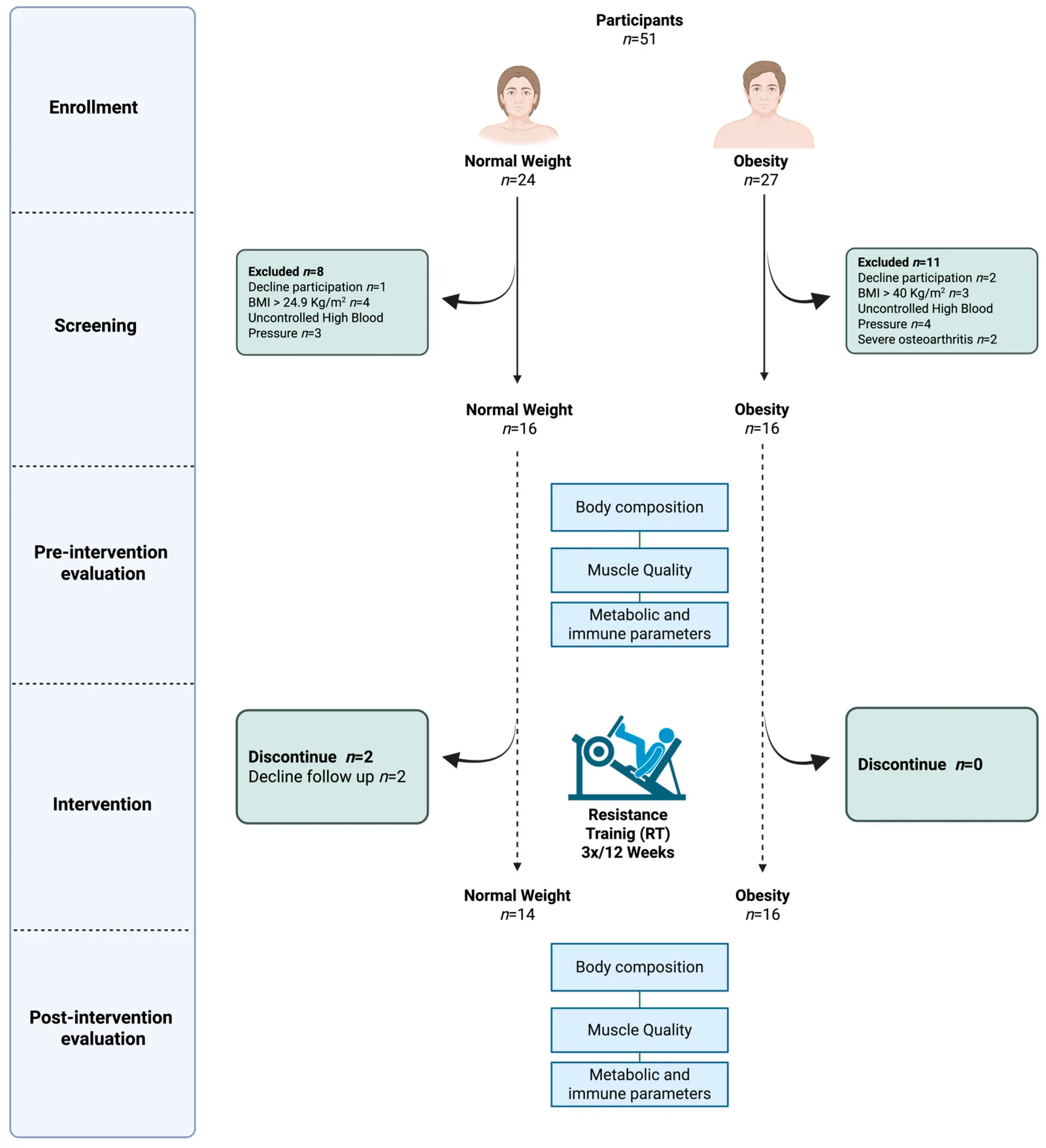
Flow diagram of study participants. A total of 51 older women were initially assessed for eligibility. Of these, 32 met the inclusion criteria and were enrolled in the study. Two participants did not complete the intervention, resulting in a final sample of 30 women, including 14 with normal-weight and 16 with obesity. Participants completed a 12-week resistance training programme consisting of three supervised sessions per week, with exercise intensity progressively increased from 60% to 80% of one-repetition maximum (1RM).

A prospective pre–post-intervention study with parallel, naturally defined groups was conducted in 30 older women who completed the study and were classified into two groups according to body composition: normal-weight women (NW: 66 ± 4 years; body mass index [BMI]: 22.60 ± 1.36 kg/m^2^; body fat percentage: 33.11% ± 3.61%; *n* = 14) and women with obesity (OB: 68 ± 4 years; BMI: 33.01 ± 2.62 kg/m^2^; body fat percentage: 42.23% ± 2.98%; *n* = 16). There was no non-intervention control group (Figure 4). This study is part of a larger research project aimed at comparing the effects of a 12-week RT programme on muscle quality, immune response, metabolic response, and physical performance in older women aged 60–79 years with obesity versus age-matched normal-weight women, as previously reported [46]. Sample size was determined using G*Power version 3.1.9.7 for comparisons between two independent groups. The calculation assumed an effect size of 0.29, a significance level of 5% (α = 0.05), and a statistical power of 95%. The expected effect size was based on changes in quadricep muscle thickness reported in a previous study conducted by our research group, which included postmenopausal women who completed the same 12-week exercise intervention and were assessed using the same ultrasound equipment [82]. Volunteers were recruited through a community-based recruitment strategy that included social media outreach, advertisements placed in different areas of Temuco, and notices displayed on bulletin boards at the Universidad de La Frontera (Chile). Participant recruitment and follow-up procedures were conducted between August 2024 and September 2025. During this period, participants underwent baseline assessments, completed the 12-week resistance training programme, and underwent post-intervention evaluations. All assessments and data collection procedures were conducted at the Universidad de La Frontera (UFRO), Temuco, Chile.

The study was approved by the Scientific Ethics Committee of the Universidad de La Frontera, Temuco, Chile (approval code No. 03/24), conducted in accordance with the Declaration of Helsinki, and registered at ClinicalTrials.gov (identifier: NCT06367296; registered 15 April 2024), as previously described [46]. All participants provided written informed consent prior to study participation [46].

One week before the study, participants completed a general health questionnaire to determine eligibility. Women aged 60–79 years were eligible for inclusion. Classification into the normal-weight (NW; BMI: 18.5–24.9 kg/m^2^) and obesity (OB; BMI: 30–39.9 kg/m^2^) groups was established using BMI and further supported by body fat percentage, waist circumference, and waist-to-hip ratio measurements to ensure a comprehensive assessment of obesity status. Exclusion criteria included participation in regular RT during the six months prior to enrolment, the presence of medical conditions incompatible with exercise training, comorbidities limiting mobility or compromising the safe performance of RT, and the use of nutritional supplements such as leucine, glutamine, casein, whey protein, or creatine.

Participants completed a supervised RT programme for 12 weeks. Assessments were conducted at two time points: 48 h before the start of the intervention (Pre) and 48 h after completion of the final training session (Post). Prior to each assessment, participants were instructed to abstain from alcohol consumption and vigorous physical activity for at least 48 h. At each assessment, fasting venous blood samples were collected to assess metabolic parameters (glucose, insulin, lipid profile, leptin, and adiponectin) and immune parameters, including cytokines (IL-1β, IL-6, IL-8, IL-10, IL-12, and TNF-α) and NETosis induction. Anthropometric and hemodynamic variables were also recorded, including body weight, height, waist and hip circumference, blood pressure, and heart rate.

Body composition (muscle mass, fat mass, and visceral fat) was assessed by bioelectrical impedance analysis, and BMI was calculated accordingly. Participants were instructed to maintain their habitual dietary intake and physical activity levels throughout the study. Dietary intake and physical activity were assessed as control variables before and after RT using a food frequency questionnaire and a 24 h dietary recall to estimate average energy and macronutrient intake, and the International Physical Activity Questionnaire—Short Form (IPAQ) along with step counts recorded over three days using a pedometer (OMRON Corporation, Kyoto, Japan), respectively. No significant changes were observed in these variables, as previously reported [46].

### 4.2. Body Composition and Muscle Quality

Absolute (kg) and relative (%) muscle mass, as well as total fat mass, were determined using bioelectrical impedance analysis (Tanita MC-980U PLUS, Tanita Corporation, Tokyo, Japan). Morphological muscle quality was assessed by measuring quadricep muscle thickness (QT), expressed as absolute (cm) and relative (%) values, using B-mode ultrasound imaging (LOGIQ™ F8, GE Healthcare, Wauwatosa, WI, USA). Measurements were performed with participants in the supine position with knees fully extended. The transducer was placed transversely at the midpoint between the anterior superior iliac spine and the superior border of the patella of the dominant lower limb, following previously described procedures [46,83]. Quadricep thickness was expressed in centimetres and as a percentage of total thigh thickness (quadriceps plus subcutaneous tissue). Functional muscle quality was evaluated by determining maximal strength (one-repetition maximum, 1RM) and relative strength (normalised to body weight) in the leg extension exercise, as well as lower-limb power using the relative sit-to-stand (STS) mean power, as proposed by Alcazar et al. (2018) [37]. Muscle mass gain following 12 weeks of RT was assessed as the percentage change in relative muscle mass and quadricep muscle thickness (dominant limb), calculated using the following formula: [(Post − Pre)/Pre] × 100. Where Pre represents the baseline value and Post the post-intervention value [46].

### 4.3. Resistance Exercise Training

The RT programme was conducted according to previously described protocols and supervised by experienced physiotherapists [46,84]. The RT intervention followed a standardised protocol previously developed by our research group. Participants completed a supervised whole-body RT programme three times per week (Monday, Wednesday, and Friday) for 12 consecutive weeks, with each session lasting approximately 60 min. Sessions consisted of a 5 min warm-up on a cycle ergometer at light-to-moderate intensity, approximately 50 min of RT, and a 5 min cool-down including global stretching exercises.

The RT programme included machine-based exercises targeting both lower and upper limbs. Lower-limb training comprised the leg press, leg extension, and leg curl, with five sets of each exercise. Upper-limb training included the chest press and elbow extension, with three sets of each exercise. All exercises were completed for 10 repetitions per set, with 2 min rest intervals between sets and exercises. Exercise order alternated between lower- and upper-limb movements to facilitate recovery and reduce accumulated fatigue. Participants completed the intervention in small groups of three individuals per session, allowing for individualised supervision while promoting interpersonal interactions among participants, thereby fostering group cohesion and enhancing adherence to the exercise programme.

Training intensity was prescribed according to one-repetition maximum (1RM) and progressed from 60% to 80% of 1RM. Specifically, participants trained at 60% of 1RM during weeks 1–2, 70% during weeks 3–4, and 80% during weeks 5–6. A new 1RM assessment was performed in week 6, after which the same progression was repeated during weeks 7–12 using the updated training loads. One-repetition maximum was directly assessed at baseline, week 6, and week 12 to determine and adjust training intensity throughout the intervention.

Training loads were individually adjusted whenever participants were able to perform more than the prescribed 10 repetitions or when the final repetitions were completed without undue effort. The movement tempo was controlled, maintaining approximately 2–3 s for both the concentric and eccentric phases of each repetition, with no pause between phases. All training sessions were supervised by a certified strength-training professional experienced in exercise prescription for older adults. Before the intervention, participants completed one familiarisation session to learn the correct exercise technique and the proper use of the equipment. Blood pressure was measured before and after every training session to ensure participant safety. Participants were required to attend at least 80% of the scheduled sessions (≥29 of 36 sessions) to be included in the final analyses.

### 4.4. Serum Collection

Blood samples were obtained after a 12 h overnight fast by venipuncture of a superficial vein in the antecubital fossa, 48 h before the first training session and 48 h after the final RT session. Six millilitres of blood was collected into tubes without anticoagulant and centrifuged at 2500 rpm for 15 min. Serum aliquots were stored in microtubes at −80 °C until further analysis.

### 4.5. Metabolic Response

The lipid profile, including serum concentrations of total cholesterol, low-density lipoprotein cholesterol (LDL-C), and high-density lipoprotein cholesterol (HDL-C), as well as fasting glucose concentrations, were determined using enzymatic colorimetric methods with an automated photometer (Metrolab 2300 Plus, Wiener Lab., Buenos Aires, Argentina). Serum insulin, leptin, and adiponectin concentrations were measured using enzyme-linked immunosorbent assay (ELISA) kits (Catalogue Nos. KAQ1251, ELH-Adiponectin-1, and RB.ELH-LEPTIN-1; Thermo Fisher Scientific Inc., Waltham, MA, USA, and RayBiotech, Inc., Norcross, GA, USA), following the manufacturers’ instructions. Insulin sensitivity was estimated using the homeostasis model assessment of insulin resistance (HOMA-IR) (glucose × insulin/405) [85].

### 4.6. Immune Response

Pro- and anti-inflammatory cytokines (IL-1β, IL-6, IL-8, IL-10, IL-12, and TNF-α) were quantified using the BD Cytometric Bead Array (CBA) Human Inflammatory Cytokines Kit (Catalogue No. 551811, BD Biosciences, San Jose, CA, USA). Data acquisition was performed on an LSR Fortessa X-20 flow cytometer (BD Biosciences), and analyses were conducted using FCAP Array Software v3.0 (BD Biosciences).

NETosis induction was assessed using live-cell imaging of human neutrophils cultured with Pre- and Post-RT serum samples for 24 h, following protocols previously described by our research group [55]. Polymorphonuclear neutrophils (PMNs) were isolated from peripheral blood of a healthy donor by density gradient centrifugation using Polymorphprep^®^ (Axis-Shield, Oslo, Norway), according to the manufacturer’s instructions. After centrifugation, the PMN-rich fraction was washed with phosphate-buffered saline (PBS) and resuspended in Ca^2+^-supplemented X-Vivo medium.

For NETosis analysis, freshly isolated PMNs were incubated with Sytox Green and seeded in 96-well plates. Cells were stimulated with centrifuged serum samples from participants (Pre and Post) and monitored for 24 h using real-time microscopy with an IncuCyte S3 system at 37 °C and 5% CO_2_. NET formation was quantified using IncuCyte software v3 2019B by measuring the fluorescent signal corresponding to extracellular DNA stained with Sytox Green, excluding fluorescence associated with other forms of cell death such as apoptosis [86].

### 4.7. Blinding and Outcome Assessment

Due to the nature of the intervention, participants and physiotherapists responsible for implementing the resistance training programme could not be blinded to group allocation. However, ultrasound measurements were performed by an independent evaluator. Likewise, blood samples were processed and analysed by laboratory personnel blinded to both group allocation and assessment time point (pre- or post-intervention). All assessments were conducted following previously described procedures.

### 4.8. Statistical Analysis

Statistical analyses were performed using IBM SPSS Statistics, version 21.0 (Corp., Armonk, NY, USA), and graphical representations were generated using Python v3.10 (Python Software Foundation, Wilmington, DE, USA). Data are presented as mean ± standard deviation (SD) and change value (Δ Post − Pre). Changes in muscle mass and quadricep muscle thickness after 12 weeks of RT are expressed as percentage change [(Post − Pre)/Pre] × 100.

Baseline comparisons between groups were performed using independent-samples *t*-tests. To examine the effects of the intervention, a repeated-measures analysis of variance (ANOVA) was conducted, with time (Pre vs. Post) as the within-subject factor and group (NW vs. OB) as the between-subject factor. Prior to the repeated-measures ANOVA, the assumptions of normality and homogeneity were assessed using the Shapiro–Wilk Test and Levene’s Test, respectively. When a significant interaction was detected, paired-samples *t*-tests were applied to assess time effects within each group, and independent-samples *t*-tests were used to compare between-group differences at Pre and Post. Effect sizes for baseline between-group comparisons were calculated using Cohen’s d. Values of d < 0.2 were interpreted as no effect, 0.2–0.49 as a small effect, 0.5–0.79 as a moderate effect, and ≥0.8 as a large effect (Cohen, 2013) [87]. For ANOVA analyses, effect sizes were estimated using partial eta squared (η^2^), with values of 0.01 indicating a small effect, 0.06 a medium effect, and ≥0.134 a large effect. Associations between quantitative variables were assessed using Pearson’s correlation coefficient. Statistical significance was set at *p* < 0.05.

## 5. Conclusions

In conclusion, older women with obesity exhibited a distinct metabolic profile at baseline compared with normal-weight older women, although systemic inflammatory status showed limited differences. After 12 weeks of resistance training, both groups demonstrated favourable adaptations in muscle mass, strength, physical performance, and selected metabolic outcomes, supporting the effectiveness of this exercise modality in promoting muscle health during ageing, regardless of obesity status. The associations between metabolic and immune biomarkers and muscle quality parameters highlight the relevance of the systemic environment in skeletal muscle adaptation, while the differential relationship between adiponectin and muscle mass according to metabolic status emphasises the complexity of interpreting this biomarker in older adults. However, these findings should be interpreted cautiously due to the relatively small sample size and exploratory nature of some associations. Further studies with larger cohorts are needed to confirm these results and elucidate the mechanisms linking resistance training with metabolic and immune regulation.

### Limitations

Although the sample size was sufficient for the primary outcome, the large number of variables analysed would have benefited from a larger sample size. Each variable was analysed independently, and formal adjustments for multiple comparisons were not applied. Therefore, although the findings are clinically relevant, they should be interpreted with caution and confirmed in future studies with larger sample sizes. Furthermore, the absence of a non-intervention control group may represent a limitation. However, the primary aim of the present study was to compare metabolic and immune responses between older women with obesity and age-matched normal-weight women following resistance training. Future studies including a non-intervention control group are warranted to better elucidate the specific effects of resistance training on these outcomes.

## Figures and Tables

**Table 1:** Participants characteristics.

	Older Normal Weight (*n* = 14)	Older Obesity (*n* = 16)	*p* Value
Age (years)	66 ± 4	68 ± 4	0.270
Waist circumference (cm)	81.54 ± 5.52	104.28 ± 7.06	**0.000**
Weight (kg)	55.66 ± 4.49	80.98 ± 9.77	**0.000**
Height (m)	1.57 ± 0.07	1.56 ± 0.06	0.902
BMI (kg·m^−2^)	22.60 ± 1.36	33.01 ± 2.62	**0.000**
Muscle mass (kg)	35.39 ± 3.38	44.16 ± 4.57	**0.000**
% Muscle mass	63.76 ± 3.04	54.68 ± 2.82	**0.000**
% Fat mass	33.11 ± 3.61	42.23 ± 2.98	**0.000**
Visceral fat (kg)	7.57 ± 1.09	12.66 ± 1.45	**0.000**
HR (b·min^−1^)	68.71 ± 7.87	73.44 ± 8.76	0.134
SBP (mm Hg)	121.57 ± 12.00	126.94 ± 9.28	0.179
DPB (mm Hg)	72.86 ± 6.41	76.56 ± 8.49	0.193
Diabetes mellitus 2 (*n*)	0	4	**0.033**
Hypertension (*n*)	4	7	0.264
Dyslipidaemia (*n*)	12	10	0.704

*n*: number of participants. BMI: body mass index; HR: heart rate; SBP: systolic blood pressure; DBP: diastolic blood pressure. Values represent means ± SD. Bold values indicated different between NW and OB at the *p* < 0.05 level.

**Table 2:** Baseline levels in metabolic and immune parameters.

	Older Normal Weight (*n* = 14)	Older Obesity (*n* = 16)	*p* Value	Effect Size	95% CI for Mean Difference		
					Mean Difference	Lower	Upper
Glucose (mg/dL )	83.59 ± 24.29	91.85 ± 15.54	0.247	0.412	−8.739	−23.865	6.386
Insulin (Ul)	6.82 ± 2.41	16.22 ± 5.69	**0.000**	2.100	−9.538	−12.859	−6.218
HOMA-IR	1.34 ± 0.47	3.77 ± 1.77	**0.000**	1.830	−2.433	−3.433	−1.433
Adiponectin (μg/mL)	19.53 ± 7.70	14.40 ± 7.57	0.052	0.671	5.792	−0.064	11.647
Leptin (ng/mL)	5.50 ± 5.62	21.96 ± 14.77	**0.001**	1.435	−16.087	−24.653	−7.521
Total cholesterol (mg/dL)	174.30 ± 38.01	183.68 ± 159.75	0.657	0.222	−6.989	−38.863	24.885
HDL (mg/dL)	64.55 ± 13.19	51.17 ± 10.40	**0.004**	1.136	13.328	4.497	22.160
LDL (mg/dL)	109.68 ± 35.93	114.34 ± 65.31	0.795	0.138	−3.225	−28.451	22.001
Triglycerides (mg/dL)	89.47 ± 42.71	145.41 ± 44.52	**0.003**	1.280	−52.334	−84.705	−19.963
IL-1b (pg/mL)	0.78 ± 0.93	0.34 ± 0.38	0.104	0.615	0.426	−0.093	0.945
IL-6 (pg/mL)	0.47 ± 0.65	1.61 ± 3.60	0.253	0.427	−1.142	−3.147	0.864
IL-8 (pg/mL)	14.25 ± 5.48	13.56 ± 8.47	0.768	0.036	0.786	−4.625	6.197
IL-10 (pg/mL)	1.08 ± 0.37	0.91 ± 0.22	0.115	0.553	0.180	−0.047	0.407
IL-12 (pg/mL)	0.74 ± 0.18	0.76 ± 0.20	0.593	0.139	−0.038	−0.183	0.107
TNF-α (pg/mL)	1.79 ± 1.16	1.44 ± 0.61	0.484	0.389	0.214	−0.405	0.833
%NETosis 6 h	2.63 ± 0.36	2.73 ± 0.54	0.549	0.223	−0.102	−0.448	0.243
%NETosis 12 h	3.23 ± 0.67	3.26 ± 1.39	0.847	0.058	0.168	−2.626	2.961
%NETosis 18 h	7.79 ± 1.72	7.73 ± 4.84	0.903	0.016	0.167	−2.626	2.961
%NETosis 24 h	14.39 ± 2.98	13.83 ± 6.56	0.765	0.107	0.574	−3.329	4.479

*n*: number of patients; HDL: high-density lipoprotein; LDL: low-density lipoprotein; IL: interleukin; values represent means ± SD; bold values indicate difference at *p* < 0.05.

**Table 3:** Effect of RT on metabolic parameters.

	Older Normal Weight (*n* = 14)			Older Obesity (*n* = 16)			Within-Subject Effects		Between-Subject Effects	*p* Δ
	Pre	Post	Δ (Post − Pre)	Pre	Post	Δ (Post − Pre)	Time	Time × Group	Group	
Glucose (mg/dL)	83.59 ± 24.29	85.55 ± 21.62	1.97 ± 9.59	91.85 ± 15.54	86.88 ± 14.65 **#**	−4.89 ± 5.67	0.298	**0.021**	0.491	**0.021**
Insulin (Ul)	6.82 ± 2.41	7.98 ± 3.30	1.17 ± 2.38	16.22 ± 5.69	17.23 ± 7.38	1.01 ± 5.22	0.164	0.918	**0.000**	0.981
HOMA-IR	1.34 ± 0.47	1.67 ± 0.78	0.33 ± 0.60	3.77 ± 1.77	3.77 ± 1.96	−0.01 ± 1.15	0.354	0.348	**0.000**	0.348
Adiponectin (μg/mL)	19.53 ± 7.70	17.23 ± 8.37	−2.30 ± 5.33	14.40 ± 7.57	13.31 ± 7.15	−1.10 ± 3.64	**0.049**	0.473	0.104	0.473
Leptin (ng/mL)	5.50 ± 5.62	5.07 ± 5.35	−0.43 ± 4.86	21.96 ± 14.77	26.11 ± 19.34	4.15 ± 9.27	0.118	0.109	**0.000**	0.109
Total cholesterol (mg/dL)	174.30 ± 38.01	165.57 ± 36.35	−8.73 ± 29.32	183.68 ± 159.75	159.75 ± 31.95	−23.93 ± 51.77	**0.047**	0.341	0.880	0.341
HDL (mg/dL)	64.55 ± 13.19	61.34 ± 11.73	−3.22 ± 5.52	51.17 ± 10.40	47.95 ± 14.34	−3.22 ± 11.75	0.071	0.999	**0.004**	0.999
LDL (mg/dL)	109.68 ± 35.93	95.12 ± 30.74	−14.56 ± 23.45	114.34 ± 65.31	107.26 ± 40.70	−7.09 ± 52.28	0.165	0.626	0.424	0.626
Triglycerides (mg/dL)	89.47 ± 42.71	79.07 ± 33.86	−10.40 ± 37.98	145.41 ± 44.52	133.03 ± 52.05	−12.38 ± 27.76	0.133	0.894	**0.001**	0.894

Pre: before RT; Post: after RT; *n*: number of patients; HDL: high-density lipoprotein; LDL: low-density lipoprotein. Values represent means ± SD; bold values indicate difference at *p* < 0.05; paired *t*-tests: # (*p* = 0.030) between Pre vs. Post.

**Table 4:** Effect of RT on immune parameters.

	Older Normal Weight (*n* = 14)			Older Obesity (*n* = 16)			Within-Subject Effects		Between-Subject Effects	*p* Δ
	Pre	Post	Δ (Post − Pre)	Pre	Post	Δ (Post − Pre)	Time	Time × Group	Group	
IL-1b (pg/mL)	0.78 ± 0.93	0.86 ± 0.75	0.08 ± 0.72	0.34 ± 0.38	0.37 ± 0.34	0.03 ± 0.39	0.601	0.807	**0.031**	0.807
IL-6 (pg/mL)	0.47 ± 0.65	1.07 ± 3.60	0.59 ± 2.24	1.61 ± 3.60	0.90 ± 1.72	−0.72 ± 3.80	0.916	0.268	0.445	0.268
IL-8 (pg/mL)	14.25 ± 5.48	14.27 ± 2.67	0.02 ± 3.95	13.56 ± 8.47	11.53 ± 6.11	−2.03 ± 4.76	0.224	0.213	0.432	0.213
IL-10 (pg/mL)	1.08 ± 0.37	1.16 ± 0.31	0.08 ± 0.42	0.91 ± 0.22	0.82 ± 0.31	−0.09 ± 0.18	0.973	0.150	**0.006**	0.150
IL-12 (pg/mL)	0.74 ± 0.18	0.87 ± 0.29	0.14 ± 0.25	0.76 ± 0.20	0.65 ± 0.15 #	−0.12 ± 0.23	0.821	**0.008**	0.126	**0.008**
TNF-α (pg/mL)	1.79 ± 1.16	2.02 ± 1.13	0.22 ± 0.56	1.44 ± 0.61	1.27 ± 0.40 #	−0.18 ± 0.44	0.785	**0.040**	0.082	**0.040**
%NETosis 6 h	2.63 ± 0.36	2.65 ± 0.39	0.02 ± 0.40	2.73 ± 0.54	2.84 ± 0.98	0.11 ± 1.11	0.684	0.796	0.402	0.796
%NETosis 12 h	3.23 ± 0.67	3.47 ± 1.25	0.24 ± 1.31	3.26 ± 1.39	4.08 ± 2.68	0.79 ± 2.07	0.111	0.402	0.532	0.402
%NETosis 18 h	7.79 ± 1.72	8.54 ± 3.62	0.75 ± 2.30	7.73 ± 4.84	9.31 ± 5.99	1.57 ± 3.73	0.054	0.482	0.482	0.482
%NETosis 24 h	14.39 ± 2.98	15.57 ± 5.64	1.18 ± 4.45	13.83 ± 6.56	16.59 ± 8.22	2.74 ± 5.56	**0.044**	0.408	0.916	0.408

Pre: before RT; Post: after RT; *n*: number of participants; IL: interleukin, TNF: tumour necrosis; values represent means ± SD; bold values indicate difference at *p* < 0.05; independent *t*-tests: # (*p* < 0.020) between Post Older Normal-Weight vs. Post Older Obesity.

## Data Availability

The datasets used and/or analysed during the current study are available from the corresponding author, Gabriel Nasri Marzuca-Nassr, on reasonable request.
